# Metformin attenuates symptoms of osteoarthritis: role of genetic diversity of Bcl2 and CXCL16 in OA

**DOI:** 10.1186/s13075-023-03025-7

**Published:** 2023-03-07

**Authors:** Nahid Alimoradi, Mohammad Tahami, Negar Firouzabadi, Elham Haem, Amin Ramezani

**Affiliations:** 1 Department of Pharmacology & Toxicology, School of Pharmacy, Shiraz University of Medical Sciences, Shiraz, Iran; 2 Bone and Joint Disease Research Center, Shiraz University of Medical Sciences, Shiraz, Iran; 3 Department of Biostatistics, Medical School, Shiraz University of Medical Sciences, Shiraz, Iran; 4 Shiraz Institute for Cancer Research, School of Medicine, Shiraz University of Medical Science, Shiraz, Iran

**Keywords:** Osteoarthritis, Metformin, Bcl-2, CXCL-16, KOOS, mTOR, Polymorphism, Double-blind placebo-controlled clinical trial

## Abstract

**Objective:**

This study aimed to evaluate the effectiveness of metformin versus placebo in overweight patients with knee osteoarthritis (OA). In addition, to assess the effects of inflammatory mediators and apoptotic proteins in the pathogenesis of OA, the genetic polymorphisms of two genes, one related to apoptosis (rs2279115 of Bcl-2) and the other related to inflammation (rs2277680 of CXCL-16), were investigated.

**Methods:**

In this double-blind placebo-controlled clinical trial, patients were randomly divided to two groups, one group receiving metformin (*n* = 44) and the other one receiving an identical inert placebo (*n* = 44) for 4 consecutive months (starting dose 0.5 g/day for the first week, increase to 1 g/day for the second week, and further increase to 1.5 g/day for the remaining period). Another group of healthy individuals (*n* = 92) with no history and diagnosis of OA were included in this study in order to evaluate the role of genetics in OA. The outcome of treatment regimen was evaluated using the Knee Injury and Osteoarthritis Outcome Score (KOOS) questionnaire. The frequency of variants of rs2277680 (A181V) and rs2279115 (938C>A) were determined in extracted DNAs using PCR-RFLP method.

**Results:**

Our results indicated an increase in scores of pain (*P* ≤ 0.0001), activity of daily living (ADL) (*P* ≤ 0.0001), sport and recreation (Sport/Rec) (*P* ≤ 0.0001), and quality of life (QOL) (*P* = 0.003) and total scores of the KOOS questionnaire in the metformin group compared to the placebo group. Susceptibility to OA was associated with age, gender, family history, CC genotype of 938C>A (Pa = 0.001; OR = 5.2; 95% CI = 2.0–13.7), and GG+GA genotypes of A181V (Pa = 0.04; OR = 2.1; 95% CI = 1.1–10.5). The C allele of 938C>A (Pa = 0.04; OR = 2.2; 95% CI = 1.1–9.8) and G allele of A181V (Pa = 0.02; OR = 2.2; 95% CI = 1.1–4.8) were also associated with OA.

**Conclusion:**

Our findings support the possible beneficial effects of metformin on improving pain, ADL, Sport/Rec, and QOL in OA patients. Our findings support the association between the CC genotype of Bcl-2 and GG+GA genotypes of CXCL-16 and OA.

## Introduction

Osteoarthritis (OA) is a multifactorial progressive degenerative disease that is associated with synovial inflammation and bone remodeling and leads to impaired functional characteristics of the joint cartilage [1]. Inflammatory mediators and factors such as cytokines and chemokines disturb the extracellular matrix (ECM) homeostasis which has a vital role in the pathogenesis of OA [2]. Development of articular cartilage degeneration leads to progressive joint space narrowing and is associated with disability and pain in the joints [3]. OA occurs most commonly in the knee joints, which severely affects the quality and quantity of life of patients [4]. The prevalence of OA significantly increases with age with higher rates in women over 45 [5].

Racial/ethnic disparities in OA are well-recognized. Several studies have shown that non-Hispanic Black (NHB) individuals experience higher rates of symptomatic and radiographic OA, higher age-related average pain intensity, and higher levels of disability than their non-Hispanic White (NHW) counterparts, in addition to higher pain sensitivity [6–8]. The role of genetics in various illnesses has been well documented [9–13]. In addition, role of genetics in OA has been supported by many studies, predominantly the single nucleotide polymorphisms (SNPs). Genetic variations could be associated with the OA progression or response to drug therapy among different ethnicities. Since different pathways and molecular mechanisms are involved in the pathogenesis of OA, various genes can affect one’s risk for developing OA [14–16].

Bcl-2 is an anti-apoptotic protein that suppresses apoptosis by inhibiting the action of Bcl-2 associated X protein (BAX), a member of the Bcl-2 family that promotes apoptosis via the mitochondrial-mediated pathway. The ratio of Bcl-2 to BAX expression indicates cellular susceptibility to apoptosis [17–19]. In a report studying the effects of an autophagy inhibitor in the induced-OA animal models, increased autophagy in cartilages of knee joints was associated with inhibition of the Akt/mTOR signaling pathway and increased Bcl-2/Bax ratio [20]. Higher level of apoptotic cartilage death in the OA involved area than in the non-involved area better reflect their role in the pathogenesis of OA [21]. In fact, a negative correlation was observed between Bcl-2/BAX ratio, aging, and OA development [22–25].

The Bcl-2 polymorphism (-938 C> A, rs2279115), a functional SNP on chromosome 18q21.33, is located in the P2 promoter region. It has been reported that the wild type of this SNP may inhibit gene transcription and thus negatively affect Bcl-2 levels [26–28]. However, no study has yet evaluated the association of the Bcl-2 rs2279115 gene polymorphism with susceptibility and severity of knee OA.

Various studies have shown that the occurrence and development of OA are closely associated with the abnormal balance between autophagy and apoptosis in the cartilage [29–31]. Recent studies have shown that the regulation of autophagy is associated with the AMPK/mTORC1 signaling pathways. Upregulation of mTOR leads to the inhibition of autophagy, while the apoptosis of OA chondrocytes is increased which plays an important role in the pathogenesis of OA [32–35]. AMPK is a regulator of cellular energy that maintains a balance between catabolism and anabolism. Decreased control of AMPK over mTOR leads to increased mTOR activity, which may eventually lead to several age-related diseases, including diabetes and OA [36–40]. Inhibition of autophagy has been shown to increase inflammatory activity and lead to increased IL-1β production, which increases mTOR expression in OA chondrocytes [41, 42].

CXCL-16 is a chemokine secreted by synovial macrophages and fibroblasts that mediates the chemo-attraction of neutrophils, lymphocytes, and monocytes into the synovium [43–45]. Soluble CXCL-16, when bound to CXCR6, activates Akt, which may, directly and indirectly, activate its downstream key protein, serine/threonine kinase, mTOR [46, 47]. In studies, serum levels of CXCL-16 were associated with areas of bone apposition/reparative proliferation in temporomandibular joint osteoarthritis (TMJOA) and chronic rheumatoid arthritis-induced inflammation [45, 48]. Studies have reported significant expression of CXCL-16 mRNA in synovial tissue and fluid of OA compared with controls [44, 49]. The CXCL-16 functional SNP, called A181V (rs2277680) on chromosome 17p13, is located in exon 4 of the gene encoding the CXCL16 stalk and encodes the A3V mutation, in which it converts alanine to valine at codon 181 (A181V), and has a strong effect on the structure of the CXCL-16 protein [50, 51]. To date, no study has evaluated the association of A181V (rs2277680) SNP with knee OA.

Metformin is a safe and tolerable oral drug known as the first-line treatment for type 2 diabetes [52]. It can also be beneficial for a number of illness from metabolic to age-related diseases due to its effect on cellular processes [53–59]. According to the role of AMPK in cartilage homeostasis, metformin as an AMPK activator can protect inflammatory cell death of chondrocytes and attenuate cartilage degeneration and development of OA, as evidenced by in vivo and in vitro results [60–63].

To date, there has been no effective non-surgical treatment for OA, and existing therapies such as analgesics and nonsteroidal anti-inflammatory drugs (NSAIDs) are only used to reduce pain and swelling. It is noteworthy that surgical therapy is the last option for patients with end-stage OA [64].

To evaluate the outcome of any therapeutic interventions for knee OA, it is recommended to use multi-item knee-specific outcome measures, such as knee injury and osteoarthritis outcome score (KOOS) to provide a broader picture of the clinical condition [65–67].

The aim of this study was to assess the effect of metformin versus placebo on symptoms of knee OA in overweight patients diagnosed with knee OA in a randomized, double-blind, placebo-controlled trial in a 4-month period using the KOOS questionnaire. Moreover, considering the role of autophagy, AMPK, and the mTOR pathways in OA as well as the effect of Bcl-2, and CXCL-16 in regulation of the mTOR pathway, we aimed to investigate the role of genetic diversity of Bcl-2 and CXCL-16 in OA.

## Methods and materials

### Participants and treatment protocol

This study was a randomized double-blind placebo-controlled clinical trial with the IRCT code of IRCT20150202020908N2. Ethical approval was obtained from the Research Ethics Committee of Shiraz University of Medical Sciences with the ethical code of IR.SUMS.REC.1398.1010. This study was conducted in accordance with the Declaration of Helsinki.

The participants were selected among the patients with knee pain referred to knee clinic of Motahari complex, Shiraz, Iran.

All patients were examined by an orthopedic knee surgeon during the first visit, and a focused physical examination and history taking were performed. Standard standing anteroposterior (AP) and lateral radiograph were taken and grade of OA was defined according to Kellgren-Lawrence (K-L) classification. Those who had grade 4 were scheduled for total knee replacement surgery. Analgesics and physical rehabilitation were prescribed for patients with K-L grade ≤ 3 who had not received any treatment prior to their referral. In case the patients were not responsive to the above-mentioned treatment protocol, they were reassessed to determine whether they are eligible to enter the study.

Inclusion criteria included BMI > 25 and grade ≤ 3 of OA according to the K-L classification. Exclusion criteria included patients < 18 years of age, cancer, autoimmune diseases, type 2 diabetes, alcoholism, inflammatory bowel disease (IBD), hepatic or renal dysfunction, previous intra-articular knee injections, and metformin use or history of use in the last 2 months. Regarding medication history, patients did not use any anti-inflammatory agent or any other drugs with anti-inflammatory properties except for NSAIDs.

All participants filled out the informed consent form prior to entry of the study. Demographic variables of the participants such as age, sex, BMI, family history, medical conditions, and drug history were collected (Table 1).

**Table 1:** Demographic data of OA patients and healthy controls

Variables	OA (*N* = 88)	Non-OA (*N* = 92)	Total (*N* = 180)	Pa (< 0.05)
**Sex,*n*(%)**				
**Female**	60 (68.2)	41 (44.6)	105 (58.3)	0.005
**Male**	28 (31.8)	51 (55.4)	75 (41.7)	
**Age (years)**	48.3 ± 9.2	33.0 ± 7.7	40.5 ± 11.4	0.0001
**BMI (kg/m**^**2**^**)**	29.1 ± 3.4	25.6 ± 4.9	27.3 ± 4.6	0.213
**Cardiovascular dx,*n*(%)**	21 (23.9)	3 (3.3)	24 (13.3)	0.284
**Family history (%)**	58 (65.9)	11 (11.9)	69 (38.3)	0.003
**Smoking**	16 (18.2)	16 (17.4)	32 (17.8)	0.890

*Pa *adjusted *P*-value, *BMI *body mass index

A total of 88 cases diagnosed with OA were enrolled in this study; OA patients were randomly divided to two groups, one group receiving metformin (*n* = 44) and the other group receiving an identical inert placebo (*n* = 44) for 4 consecutive months (starting dose 0.5 g/day for the first week, increase to 1 g/day for the second week, and further increase to 1.5 g/day for the remaining period).

Allocation of patients was based on a simple randomization method of two sets of envelopes with the name of the drug/placebo prescribed inside. Envelopes were shuffled, and the clinician sequentially opened the envelopes to determine the treatment group for each patient. Both the prescribing physician and the patients were blinded to the treatment as well as the analyst who measured the response rate.

In order to assess the possible association of genetic variants of Bcl-2 and CXCL-16 and OA, healthy people with no history of disease were invited to enroll the study by a public announcement at Motahari Medical Complex. Absence of OA was confirmed by physical examination, use of the KOOS questionnaire, and complementary radiologic evaluation when there was a doubt. Finally, 92 healthy non-OA participants were included as a negative control group.

### Questionnaire

KOOS, a valid and reliable questionnaire that has been developed for use in practice and research to evaluate the short-term and long-term outcomes of knee injury such as osteoarthritis, was used to assess the effect of treatment on OA outcome. The questionnaire comprises 42 items in 5 subscales which include pain, other symptoms, function in daily living [3], function in sport and recreation (Sport/Rec), and knee-related quality of life (QOL) [65]. The participants completed the paper versions of the KOOS at the beginning and at the end of the study period. All items were scored on a scale of 0 to 4 and summed, where 0 indicates no difficulty and 4 indicates severe difficulty. The initial raw data from each subscale was converted to a scale from 0 to 100 (worst to best) [68].

### Genotyping

Alongside, another group of healthy individuals (*n* = 92) with no history and diagnosis of OA was included in this study in order to evaluate the role of genetics in OA. Blood sample was collected from each participant in an EDTA tube, and genomic DNA was extracted from whole blood leukocytes by the salting out extraction method previously described [69]. The quality of extracted DNA was assessed using NanoDrop®, which ensured high-quality DNA for subsequent genotyping experiments. The detailed sequence information of the study SNPs is mentioned in Table 2. Analysis of SNP of polymorphism rs2277680 and rs2279115 were carried out via the polymerase chain reaction-restricted fragment length polymorphism (PCR-RFLP) method. Genotyping of both SNPs was carried out with a slight modification of previously described protocols [51, 70]. A total of 50 ng genomic DNA was mixed with 0.2 pmol of each PCR primer in a total volume of 25 μl containing 12.5 μl Master Mix (2X) (Ampliqon, Denmark). PCR cycling conditions were as follows: an initial denaturation at 95 °C for 5min, followed by 35 cycles at 95 °C for 40–45 s, 64 °C for 40 s, and 72 °C for the 30s (rs2277680), and 30 cycles at 95 °C for 45 s, 58°C for 40 s, 72 °C for the 30s (rs2279115), and a final extension at 72 °C for 7 min (rs2277680) and 72 °C for 10 min (rs2279115). After PCR amplification, PCR products of rs2277680 and rs2279115 polymorphisms were incubated with restriction enzymes PvuII (Fermentas, Lithuania) at 37 °C, for 16 h, and BccI (New England Biolabs, England) at 37 °C for 1 h. Digested fragments were separated by electrophoresis on a 3% agarose gel (Invitrogen® Ultra-Pure, Waltham, Massachusetts) and visualized in a UV transilluminator.

**Table 2:** List of forward and reverse primers for PCR-RFLP of rs2277680 and rs2279115 and their associated restriction enzymes and DNA fragments

Polymorphism	Primer sequence (5′-3′)	TA ^(°C)^	Restriction enzyme	DNA fragment size (bp)	References
938C>A (rs2279115)	F-CTGCCTTCATTTATCCAGCA R-GGCGGCAGATGAATTACAA	58 °C	BccI 37°C/1h	300/189/111	[1]
A181V (rs2277680)	F-CAGAAGCATTTACTTCCTACCAG R-ACTGTGGCTGATGTCCTGGCT	64 °C	PvuII 37°C/16h	281/206/75	[2]

*TA* annealing temperature, *bp* base pair

### Statistical analysis

Data were analyzed using the SPSS® 21.0 for Windows (SPSS Inc., Chicago, Illinois) software. Due to a number of dropouts in the metformin group, an intent-to-treat analysis was performed. Continuous variables are demonstrated as mean ± SD, and categorical variables are presented in percentages (%). Mann-Whitney test was used to analyze non-parametric data, while *t*-test was used for parametric data analysis. In order to rule out the false positive issue in the analysis of the KOOS subscales, *T*^2^-Hotelling multivariate test was performed. Gene-disease association was assessed using odds ratio (OR) and 95% confidence interval (CI) estimation based on the following genetic contrast/models: (1) allele contrast (C-allele vs A-allele, and G-allele vs A-allele); (2) recessive model (CC vs CA+AA and GG vs GA+AA); (3) dominant model (CC+CA vs AA and GG+GA vs AA) for the genetic polymorphisms of the two SNPs, rs2279115 of Bcl-2 and rs2277680 of CXCL-16, respectively. Distributions of genotypes was calculated by chi-square (*χ*^2^) test. Adjusted *P*-values were calculated using multiple logistic regression analysis. *P*-value < 0.05 was considered as statistically significant.

## Results

A total of 88 patients diagnosed with OA based on KOOS scale were enrolled in our randomized clinical trial. Ninety-two healthy participants were recruited as well. Twenty-seven patients in the metformin group and 44 patients in the placebo group completed the study. The drop-out in the metformin group was because of lack of tolerance due to side effects of metformin such as dizziness, lightheadedness, lethargy, or digestive problems which occurred by increase in the dosage of the drug up to 1500 mg/day.

All the enrolled participants completed the KOOS questionnaire (Table 1). Among the total of 180 enrolled participants, 44 were metformin users (age, 49.3 ± 9.3 years; 77.3% female, 22.7% male), 44 were placebo users (age, 47.3 ± 9.0 years; 68.2 female, 31.8 male), and 92 were healthy controls (age, 33.0 ± 7.7 years; 44.6 female, 55.4 male). The mean BMI of the metformin, placebo, and healthy control group were 29.1 ± 3.0 kg/m^2^, 29.0 ± 3.8 kg/m^2^, and 25.6 ± 4.9 kg/m^2^ respectively.

In terms of age, gender, and family history, there was a significant difference between OA patients (metformin and placebo) and healthy controls (*P* ≤ 0.001, *P* ≤ 0.005, and *P* ≤ 0.003, respectively) (Table 1), while no significant difference was observed between metformin and placebo groups (*P* ≥ 0.05) regarding these parameters (Table 3). Also, there was no significant differences between OA patients and healthy control regarding smoking status (*P* = 0.890). Additionally, the use of metformin for 4 months did not cause significant differences in BMI or weight change between participants in metformin group (*P* = 0.951).

**Table 3:** Demographic data of metformin and placebo groups

Variables	Metformin (*N* = 44)	Placebo (*N* = 44)	Total (*N* = 88)	Pc (< 0.05)
**Sex,*n*(%)**				
**Female**	34 (77.3)	30 (68.2)	64 (72.7)	0.344
**Male**	10 (22.7)	14 (31.8)	24 (27.3)	
**Age (years)**	49.3 ± 9.3	47.2 ± 9.0	48.3 ± 9.2	0.277
**BMI (kg/m**^**2**^**)**	29.1 ± 3.1	29.0 ± 3.8	29.1 ± 3.4	0.828
**Cardiovascular dx,*n*(%)**	13 (29.5)	8 (18.2)	21 (23.9)	0.494
**Family history (%)**	30 (63.6)	32 (72.7)	62 (70.5)	0.447
**K-L grade,*n*(%)**	16 (36.4)			
**1**		20 (45.5)	36 (40.9)	0.229
**2**	21 (47.7)	22 (50)	43 (48.9)	
**3**	7 (15.9)	2 (4.5)	9 (10.2)	

*Pc* *P*-value for chi-square test, *BMI *body mass index

The mean baseline KOOS scores of patients in metformin, placebo, and healthy control groups were 52.7 ± 18.4, 52.7 ± 19.0, and 86.8 ± 10.0, respectively. There was a significant difference between symptoms, pain, ADL, Sport/Rec, and QOL scores in patients with OA and the healthy control group at baseline (*P* ≤ 0.0001), as expected. The mean total KOOS scores of patients in the metformin and placebo group at the end of the study were 65.8 ± 19.8 and 51.0 ± 18.2, respectively (*P* ≤ 0.0001), while there was no significant difference in the mean of KOOS scores between the two groups prior to intervention (*P* ≥ 0.05) (Table 4). Treatment with metformin resulted in a significant improvement in pain, ADL, Sport/Rec, and QOL scores of the KOOS questionnaire at the end of the study compared to the pre-treatment time (time 0) within the same group (*P* ≤ 0.0001) (Table 4).

**Table 4:** The KOOS scores pre and post-treatment in metformin and placebo groups

KOOS scales	Mean ± SD		Pm	Mean ± SD		Pp	P mp
	Metformin (***N*** = 44)			Placebo (***N*** = 44)			
	Pre-treatment	Post-treatment		Pre-treatment	Post-treatment		Post-treatment
Total	52.7 ± 18.4	65.8 ± 19.8	0.0001	52.7 ± 19.0	51.0 ± 18.2	0.055	0.0001
Symptoms	77.7 ± 14.9	78.7 ± 17.4	0.664	75.6 ± 17.3	73.6 ± 17.5	0.014	0.166
Pain	54.9 ± 18.8	70.8 ± 19.1	0.0001	51.8 ± 19.0	50.5 ± 19.0	0.07	0.0001
ADL	55.7 ± 20.8	72.9 ± 19.2	0.0001	55.1 ± 24.1	56.3 ± 20.4	0.362	0.0001
Sport/Rec	35.1 ± 29.0	53.0 ± 31.3	0.0001	37.8 ± 28.6	32.3 ± 23.7	0.001	0.0001
QOL	39.6 ± 20.3	53.6 ± 26.0	0.002	41.1 ± 20.5	41.6 ± 21.6	0.472	0.003

*Pm*, the calculated *P* value between the pre and post-treatment times in the metformin group; *Pp*, the calculated *P* value between the pre and post-treatment in placebo group; *P mp*, the calculated *P* value between post-treatment times of the metformin and placebo groups

In detail, a significant increase in the item of swelling in symptoms score was observed in the metformin group between time zero and the end of the study (*P* = 0.006); however, in the total score of symptoms, no significant difference was observed. Treatment with placebo resulted in a reduction in the symptoms and Sport/Rec scores compared to the pre-treatment values (*P* = 0.014, and *P* ≤ 0.001, respectively). There was no significant difference in all calculated KOOS scores in metformin and placebo groups at zero time (*P* ≥ 0.05), while after treatment, all scores except for symptom scores were significantly different, and the beneficial effects of metformin treatment compared to placebo treatment were clear (*P* ≤ 0.05) (Table 4). The *P*-value of *T*^2^-Hotelling test was < 0.001. As expected, the scores of metformin users at the end of the study were still significantly different from the healthy subjects (*P* ≤ 0.0001).

Genotype and allele distribution of Bcl-2 and CXCL-16 gene polymorphism are presented in Table 5. *P*-values after adjusting for confounders (age, sex, and family history) are provided as Pa. As shown, for Bcl-2 gene, significant association with OA was found in a recessive model (Pa = 0.001; OR = 5.2; 95% CI = 2–13.7 for CC vs CA+AA). For CXCL-16 gene, significant association with OA was found in a dominant model (Pa = 0.04; OR = 2.1; 95% CI = 1.1–10.5 for GG+GA vs AA).

**Table 5:** Genotype and allele distribution in OA patients and healthy controls

SNP		Frequencies (%)			Pc	OR; 95%CI	Pa	OR; 95%CI
	Subjects	OA		Control (*n* = 92)				
		Metformin (*n* = 44)	Placebo (*n* = 44)					
	**Genotype**							
938C>A (Bcl-2)	CC	21 (47.7)	28 (63.6)	28 (30.4)	0.0008	2.9; 1.9–5.4	0.001	5.2; 2.0–13.7
	CA	15 (34.1)	12 (27.3)	54 (58.7)				
	AA	8 (18.2)	4 (9.1)	10 (10.9)				
	**Allele**							
	C	57 (64.8)	68 (77.3)	110 (59.8)	0.02	1.7; 1.1.–2.6	0.04	2.2; 1.1–9.8
	A	31 (35.2)	20 (22.7)	74 (40.2)				
	**Genotype**							
A181V (CXCL-16)	GG	20 (45.5)	20 (45.5)	28 (30.5)	0.007	3.4; 1.4–8.5	0.04	2.1; 1.1–10.5
	GA	19 (43.2)	22 (50.0)	43 (46.7)				
	AA	5 (11.3)	2 (4.5)	21 (22.8)				
	**Allele**							
	G	59 (67.0)	62 (70.5)	99 (53.8)	0.004	1.9; 1.2–2.9	0.02	2.2; 1.1–4.8
	A	29 (33.0)	26 (29.5)	85 (46.2)				

*Pc P*-value for chi-square test, *Pa* adjusted *P*-value, *OR* odds ratio, *CI* confidence interval

The C allele of 938C>A (Pa = 0.04; OR = 2.2; 95% CI = 1.1–9.8) and G allele of A181V (Pa = 0.02; OR = 2.2; 95% CI = 1.1–4.8) were associated with OA.

## Discussion

In this study, we conducted a double-blind placebo-controlled clinical trial to determine whether metformin intervention can affect OA, ameliorate signs, and pain or modify activity in overweight non-diabetic knee OA patients within 4 months of treatment. We also assessed the role of genetic variations of Bcl-2 and CXCL-16 in OA. The results of our study demonstrated significant beneficial effects of metformin on the general conditions of OA patients. Metformin also caused a significant reduction in knee swelling. On the other hand, placebo treatment did not produce beneficial effects on the condition of OA patients.

The results of another clinical trial in OA patients confirm the beneficial effects of metformin such as changes in western Ontario and McMaster universities osteoarthritis index (WOMAC) score and visual analog scale (VAS) knee pain with a maximum dose of 2 g/day for 24 months [71]. In another study, the results showed that metformin (1000 mg/day for 12 weeks) in combination with meloxicam (15 mg/day) significantly improved KOOS components compared to the setting that meloxicam was used alone in OA patients [72]. Recently, some studies have reported the therapeutic influence of metformin on reducing cartilage degeneration and joint pain in animal models and cohort studies [73–76]. In our study, with pain reduction following metformin administration, pain consequences such as a disability in activities of daily living, exercise, physical reactions, and quality of life also improved in OA patients. In this regard, the effects of metformin in reducing pain and edema which was observed in our study can be attributed to many factors, including its anti-inflammatory and antioxidant effects [77–79]. mTOR signaling has been reported to sensitize the nervous system in conditions such as chronic pain along provoking inflammation [80, 81]. Additionally, the role of AMPK in reduction of pain and inflammation is clear. As known, metformin inhibits the mTOR pathway and its downstream pathways by enhancing AMPK phosphorylation. It also maintains the balance between apoptosis and autophagy of chondrocytes and other cells by activating AMPK and reduces cartilage degeneration by affecting the mTOR pathway and its downstream pathways [39, 61–63, 74]. As a result, metformin reduces pain and edema by inhibiting apoptosis, reducing oxidative stress, and increasing autophagy [79, 81–83].

Other findings of our study were that the carriers of CC genotype of 938C>A were significantly more prone to OA. Bcl-2 gene consists of two promoters, P1 and P2; the P2 promoter plays a regulatory role on the P1 promoter and reduces its activity. The SNP 938C>A is located on the P2 promoter. Previous studies suggest that the C allele increases the activity of the P2 promoter and thus inhibits gene transcription by the P1 promoter and promotes apoptosis by reducing Bcl-2 gene expression [26–28]. The A allele is reported to be associated with increased cancer risk by increasing the expression level of Bcl-2 and inhibiting apoptosis [28, 84–86]. According to different studies, Bcl-2 mRNA expression level is significantly decreased in the cartilage tissue of patients with high degree of OA or in animal models; however, in other studies, this reduction of Bcl-2 level was not supported statistically [22, 87–89]. The only SNP studied previously in OA patients is the Bcl-2 -717 C>A polymorphism where its homozygous mutant genotype was reported to be associated with decreased expression of Bcl-2, promotion of apoptosis, and development of OA [90]. Bcl-2 is one of the important elements of interaction between the apoptotic and autophagy systems [91]. It has been reported that binding of the autophagy protein Atg4B to Bcl-2 leads to degradation of Bcl-2-Beclin-1 and induces Bcl-2-Beclin1 dissociation. The release of Beclin-1 and the subsequent binding to PI3KC3 regulates autophagy initiation which finally leads to switch from apoptosis to autophagy [92–94]. According to the effect of CC genotype in increasing apoptosis by reducing the production of Bcl-2, the probability of developing OA may increase in individuals carrying this genotype [22, 88, 90].

Regarding CXCL16 genetic variant, A181V, our results advocate the association between GG+GA genotypes as well as its associated allele, the G allele, and OA. Missense mutation of rs2277680 (A181V) causes G→A substitutions at positions 4585312 on chromosome 17, resulting in an alanine to valine substitution at codon 181 (A181V) of the CXCL-16 protein which is at its mucin-type stalk region [51]. This substitution is located in the interior of the CXCL-16 protein and affects the conformation of the chemokine domain and reduces the size of its active site, which is associated with a loss of CXCR6-mediated adhesion in human monocytes and chemotactic activity [51, 95]. The presence of this mutation was reported to be associated with an increased risk for the development of sepsis and multiple organ dysfunction syndrome (MODS) in patients with major trauma as well as acute liver failure (ALF) in patients with Hepatitis B virus (HBV) infection, as a result of lack of natural killer T (NKT) cells activation [95]. CXCL-16 is a pro-inflammatory cytokine with angiogenic properties which stimulates synovial neovascularization [96, 97]. In chronic joint inflammation of OA, the balance between pro- and anti-inflammatory cytokines is tilted towards pro-inflammatory cytokines, which ultimately leads to the initiation of angiogenesis and bone remodeling [98, 99]. Neovascularization plays an important role in the pathology of OA, and its degree is related to the degree of OA [100, 101]. The formation of new vessels, in turn, leads to increased leukocyte recruitment and migration of neurons to the joint. These new neurons are sensitized to pain through mechanical stress, hypoxia, and inflammation. Finally, the stimulation of synovial neovascularization leads to the recurrence of pain and inflammation in the joint and exacerbation of OA [102, 103]. One study demonstrated that CXCL-16/CXCR6 chemokine signaling is associated with invasive growth and angiogenic activities in PCa cells by inducing the activation of Akt/mTOR pathways [104].

The G allele of A181V has been reported to result in the production of CXCL-16 protein with an effective protein-receptor interaction site (CXCL-16/CXCR6). This effective binding increases synovial inflammation and neovascularization induced by CXCL-16 in individuals carrying these genotypes, which eventually leads to the development of OA [95, 97, 98].

It is noteworthy that metformin modulates Beclin1–Bcl-2 complex dissociation, which regulates initiation of autophagy leading to switch from apoptosis to autophagy in an AMPK-dependent and AMPK-independent manners. In the AMPK-dependent manner, metformin phosphorylates MAPK8 which mediates Bcl-2 phosphorylation and in the AMPK-independent manner metformin inactivates STAT3, an upstream protein of Bcl-2 [105–107]. Alternatively, metformin can inhibit mTOR phosphorylation leading to the activation of ULK1, which phosphorylates Beclin-1, thus enhancing Beclin-1-PI3KC3 complex formation and autophagy induction [80, 108]. Moreover, it has been reported that metformin decreases the mRNA expression and plasma levels of CXCL-16 (108-110). Accordingly, if our study be replicated in a larger population of patients with OA, it is possible that patients respond in different degrees to metformin. Hence, the role of studied variants of Bcl-2 and CXCL-16, as targets of metformin, in response to metformin treatment can be assessed in future studies.

As the study limitation, we should allude to the relatively small sample size of the enrolled subjects. However, positive results showing the beneficial effects of somewhat short-term treatment of OA in non-diabetic patients opens up a venue to explore the effect of metformin in larger sample sizes. Alongside, the association between genetic variants of Bcl-2 and CXCL-16 and OA was investigated for the first time which may provide preliminary insights for further investigations in various ethnicities and larger study groups.

## Conclusion

A 4-month period of treatment of non-diabetic OA patients with metformin provided significant beneficial effects. Due to good safety profile and limited and mostly tolerable side effects of metformin, this oral hypoglycemic agent may be considered as an alternative or adjuvant treatment of OA patients. Additionally, significant associations with OA susceptibility were observed in carriers of different variants of 938C>A and A181V polymorphisms. We identified a possible risk genotype, wild genotype (CC) of Bcl-2 and wild allele (G) of CXCL-16 for OA susceptibility. Assessing patients more at risk of OA based on their genetic pattern may provide clues for considering preventive options in patients more at risk.

## Data Availability

The datasets used and/or analyzed during the current study are available from the corresponding author on reasonable request.

## Abbreviations

OA: Osteoarthritis
KOOS: Knee Injury and Osteoarthritis Outcome Score
ADL: Activity of daily living
Sport/Rec: Sport and Recreation
QOL: Quality of life
ECM: Extracellular matrix
NHB: Non-Hispanic Black
NHW: Non-Hispanic White
SNPs: Single nucleotide polymorphisms
BAX: Bcl-2 associated X protein
TMJOA: Temporomandibular joint osteoarthritis
NSAIDs: Non-steroidal anti-inflammatory drugs
K-L: Kellgren-Lawrence
IBD: Inflammatory bowel disease
BMI: Body mass index
PCR: Polymerase chain reaction
PCR-RFLP: PCR--restricted fragment length polymorphism
OR: Odds ratio
CI: Confidence interval
WOMAC: Western Ontario and McMaster universities osteoarthritis index
MODS: Multiple organ dysfunction syndrome
ALF: Acute liver failure
HBV: Hepatitis B virus
NKT: Natural killer T
IRCT: Iranian Registry of Clinical Trials
